# Usefulness of Liquid Biopsy Biomarkers from Aqueous Humor in Predicting Anti-VEGF Response in Diabetic Macular Edema: Results of a Pilot Study

**DOI:** 10.3390/jcm8111841

**Published:** 2019-11-02

**Authors:** Patricia Udaondo, Cristina Hernández, Laura Briansó-Llort, Salvador García-Delpech, Olga Simó-Servat, Rafael Simó

**Affiliations:** 1 Department of Ophthalmology, Hospital Universitari i Politècnic La Fe, 46009 Valencia, Spain; patyudaondo@hotmail.com (P.U.); salvadorgarciadelpech@gmail.com (S.G.-D.); 2 Diabetes and Metabolism Research Unit, Vall d’Hebron Research Institute, 08035 Barcelona, Spain; cristina.hernandez@vhir.org (C.H.); laura.brianso@vhir.org (L.B.-L.); olga.simo@vhir.org (O.S.-S.); 3 Universitat Autònoma de Barcelona, 08193 Barcelona, Spain; 4 Centro de Investigación Biomédica en Red de Diabetes y Enfermedades Metabólicas Asociadas (CIBERDEM), Instituto de Salud Carlos III (ICSIII), 28029 Madrid, Spain

**Keywords:** diabetic macular edema, anti-VEGF agents, aqueous humor, biomarkers, VEGF, inflammation, personalized medicine

## Abstract

The objective was to investigate the usefulness of the “liquid biopsy” of aqueous humor (AH) to predict the clinical response after intravitreal injections (IVT) of anti-VEGF agents for treating diabetic macular edema (DME). For this purpose, AH samples obtained during the first anti-VEGF IVT from 31 type two diabetic patients were analyzed. Patients were classified into three groups according to their anti-VEGF response: rapid responders (*n* = 11), slow responders (*n* = 11), and non-responders (*n* = 9). In addition, patients (*n* = 7) who showed good response to corticosteroids but a delayed or no response to anti-VEGF therapy were analyzed. Levels of 17 different cytokines, chemokines, and growth factors in AH were measured using a multiplex immunoassay. We found higher concentrations of VEGF in rapid responders to anti-VEGF therapy compared to non-responders. In addition, slow responders to anti-VEGF treatment showed higher levels of inflammatory markers than rapid responders, but did not reach statistical significance. Finally, those patients who responded to corticosteroids but not to anti-VEGF therapy showed significantly lower levels of VEGF than patients with rapid response (*p* = 0.01). In conclusion, “liquid biopsy” of AH could be useful to determine whether the predominant pathogenic event is primarily angiogenic or inflammatory in nature. This approach would allow physicians to select a more rational and cost-effective treatment. Further studies to validate these preliminary results are warranted.

## 1. Introduction

Diabetic retinopathy (DR) prevalence in the diabetic population is around one-third, and one-tenth has vision-threatening states such as diabetic macular edema (DME) or proliferative diabetic retinopathy (PDR) [1]. While PDR is the most common sight-threatening complication in type one diabetes, DME is the primary cause of poor visual acuity in type two diabetes [2]. Because of the high prevalence of type two diabetes, DME is the main cause of visual impairment in diabetic patients. In addition, DME is almost invariably present when PDR is detected in type two diabetes [3].

The hallmark feature of DME is the alteration of the blood-retinal barrier (BRB). Inflammation plays a crucial role in DME with the involvement of several chemokines and cytokines, including vascular endothelial growth factor (VEGF) [4,5,6]. Intravitreal anti-VEGF agents have emerged as new treatments for the more advanced stages of DR [7,8,9]. In recent years, the results of major randomized controlled clinical trials investigating the use of anti-VEGF therapy for DME have been reported. These trials provide robust evidence that intraocular administration of anti-VEGF agents is better than laser therapy both in preserving and in improving vision for patients with DME. However, around 50% of DME patients did not adequately respond to anti-VEGF therapy [5]. These findings strongly suggest that other molecules and mechanisms may operate independently or in conjunction with VEGF in the pathogenesis of this disease, with proinflammatory cytokines being one of the most significant players [5]. In the classical study of Aiello et al. [10], more than half of the patients with PDR did not show increased VEGF levels in the vitreous fluid. Therefore, it is not surprising that approximately half of patients with DME do not show adequate response to anti-VEGF treatment. In this subgroup of non-responders other growth factors and/or proinflammatory cytokines unrelated to VEGF probably play a more relevant pathogenic role. 

Proteins secreted by the retina enter the vitreous and, therefore, concentration levels of particular proteins in the vitreous could be used as markers of the function or dysfunction of the retina [11]. Furthermore, most of the proteins secreted into the vitreous diffuse into the aqueous humor (AH) which fills the anterior chamber. Since the concentration of a protein in AH may reflect its level in the vitreous and the retina [12,13], the analysis of the AH obtained at the time of the first intravitreal injection could be useful for examining whether the predominant pathogenic event is inflammation or the increase of VEGF or other growth factors. This approach would be useful for selecting a more rational and probably a most cost-effective treatment for DME [14]. However, data available on liquid biopsy of AH as a tool for predicting the response to anti-VEGF treatment are scarce and sometimes conflictive [15,16]. 

The main aim of the present study was to investigate the usefulness of a panel of cytokines and growth factors obtained from AH of patients with DME as predictors of the clinical response to intravitreal injections of anti-VEGF agents. In addition, the correlation between vitreous and aqueous levels of VEGF was examined.

## 2. Experimental Section

### 2.1. Patients

This was a retrospective study comprising a total of 31 consecutive type two diabetic patients who received anti-VEGF intravitreal injections (either ranibizumab (25% of patients) or aflibercept (75%)) for the treatment of DME between January 2017 and June 2018 in a tertiary hospital. A subset of type two diabetic subjects (*n* = 7) with a good response to intravitreal corticosteroids (dexamethasone implant) but with incomplete response to anti-VEGF therapy was also analyzed. In all cases, the sample of AH was obtained at the first intravitreal injection of anti-VEGF. All enrolled patients gave informed consent for the procedure and for entering the study. 

Apart from ophthalmologic data (i.e., best-corrected visual acuity (BCVA), funduscopic examinations and spectral-domain optical coherence tomography (SD-OCT) assessments), the clinical variables obtained in all patients included in the study were as follows: age, gender, duration of diabetes, presence and degree of DR, metabolic control (HbA1c values), and any other systemic or ocular treatment received during the follow-up. The duration of the study was 18 months. 

A volume of ≈ 0.1 mL of AH was obtained using a limbal approach with a 30 gauge needle. In 7 out of 38 patients, 1–2 mL of vitreous fluid was also obtained during 23 gauge pars plana vitrectomy [17]. The samples were immediately transferred to sterile plastic tubes, frozen, and stored at −80° until the analyses.

The study was conducted according to the Declaration of Helsinki and was approved by the Ethics Committee of Vall d’Hebron University Hospital.

### 2.2. Definition of Response to Treatment

The response to corticosteroid treatment with dexamethasone intravitreal implant can be generally established after the first injection and in the present study it was defined as a ≥50% reduction in central retinal thickness (CRT) at 6–8 weeks [18]. Conversely, evidence shows that the response to anti-VEGF therapy could not appear after the first injection [19] and, therefore, it was decided to wait to after a period of loading doses (3–5 monthly intravitreal administrations) to classify the response into three categories:

(1) Rapid response: reduction >50% in CRT at three months (one month after the third injection);
(2) Slow response: reduction between 10–50% in CRT at three months, but >50% at five months (one month after the fifth injection);
(3) No response: <20% reduction in CRT at both three and five months [20].

It should be noted that reductions in CRT are not referred from baseline, but reduction of >50% of excess CRT (defined as CRT difference from baseline and 250 µm).

### 2.3. Aqueous and Vitreous Humor Measurements

Analyses were performed with MILLIPLEX^®^ Multiplex Assays (Merck-Millipore, Darmstadt, Germany) using a Luminex-MAGPIX^®^ platform (Luminex Corporation, Austin, TX, USA). Levels from 17 factors were measured by using the following two panels: HCYTOMAG-60K-10 (EGF, IL-1β, IL-6, IL-8, IL-10, IL-12p70, PDGF-AA, PDGF-AB/BB, TNF-α, VEGF-A) and SPR1284 (PlGF, angiopoetin-2, HGF, sTNFR-I, sTNFR-II, MMP-9).

The coefficient of intra-assay variation and lower detection limits are displayed in Table 1.

### 2.4. Statistical Analyses

Comparisons between groups were made using the Student’s t test for continuous variables and the χ2 test for categorical variables. The relationship between the continuous variables was examined by Spearman’s rank correlations. All p values were based on a two-sided test of statistical significance. Significance was accepted at the level of *p* < 0.05. Statistical analyses were performed using the SSPS statistical package (SPSS, Chicago, IL, USA).

## 3. Results

The baseline characteristics of diabetic patients treated with anti-VEGF agents included in the study are specified in Table 2. The three groups were balanced regarding metabolic control (HbA1c), central retinal thickness and macular volume SD-OCT parameters. 

Regarding diabetic retinopathy (DR) progression, none of the patients experienced worsening and 35% improved at least two steps in the DR severity scale compared to baseline. The improvement of DR was independent to the response of the macular edema.

**Figure 1:**
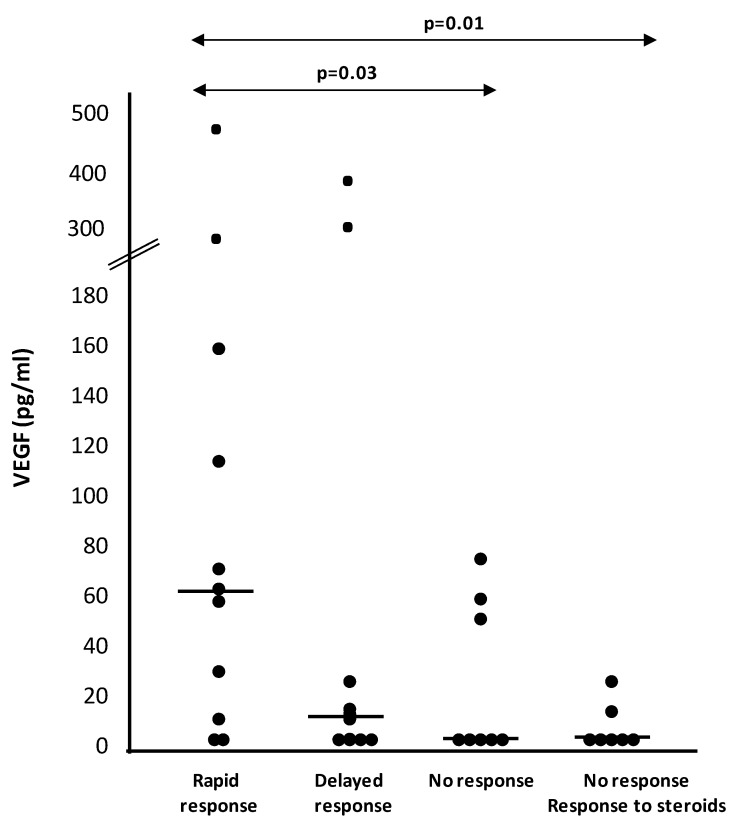
VEGF levels in aqueous humor according to response. Individual data points, median (horizontal bar symbols) and p values are shown.

**Figure 2:**
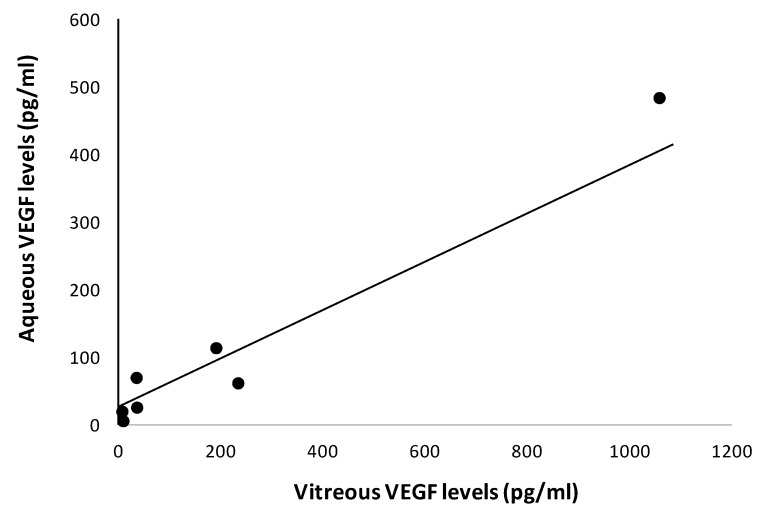
Correlation between aqueous and vitreous levels of VEGF.

The results of all the factors analyzed according to the response are summarized in Table 3. We found significantly higher levels (pg/mL) of VEGF in AH in rapid responders vs. non-responders (62.2 pg/mL (10–160) vs. 2.7 pg/mL (2.7–55); *p* = 0.03). In addition, a tendency (*p* = 0.08) towards showing higher AH VEGF levels in rapid vs. slow responders was observed. Conversely, in those patients who responded to corticosteroids but not to anti-VEGF agents, we found significantly lower levels of VEGF in comparison with rapid responders (Figure 1). Notably, we found a strong correlation between VEGF concentrations in vitreous and AH (*r* = 0.81; *p* < 0.05) (Figure 2).

Levels of IL-1β, IL-10, IL-12, EGF, TNF-α, and PDGF-AB/BB were found to be undetectable in most samples. Therefore, only PlGF, HGF, angiopoietin-2, PDF-AA, MMP-9, IL-6, IL-8, TNFR1 and TNFR2 were analyzed. Slow responders to anti-VEGF therapy showed higher levels of inflammatory markers (IL-6, IL-8, TNFR1, TNFR2 and MMP-9) than rapid responders, but these differences did not reach statistical significance (Table 3). 

## 4. Discussion

In the present study, we found significantly higher baseline levels of VEGF in the AH of the rapid responders group, in comparison with non-responders to anti-VEGF treatment. In addition, those patients who responded to corticosteroids but not to anti-VEGF agents showed significantly lower levels of VEGF than patients with rapid response. These results point to AH ‘liquid biopsy’ as a potentially useful tool for decision making regarding DME treatment selection based upon pathophysiological reasons. 

Several studies have investigated the predictive value of AH analysis in the DME treatment response. However, the results of those studies show substantial heterogeneity and variability. On the one hand, Shimura et al. [15] showed that good responders to ranibizumab presented significantly increased baseline AH VEGF concentrations. In contrast, Hillier et al. [16] reported that elevated baseline AH VEGF levels were associated with a less favorable SD-OCT macular volume response to ranibizumab. Those conflicting results could be explained by differences in the characteristics of patients included and the definition of response. For instance, in the study of Hillier et al. [16], both type one and type two diabetic patients were included and the mean levels of HbA1C were higher than 8%, whereas in Shimura’s paper [15], as well as in the present study, only type two diabetic patients were included and glycemic control was better (a mean of HbA1c lower than 8%). Unfortunately, the impact of blood glucose control in the efficacy of anti-VEGF therapies for DME was not evaluated in the pivotal clinical trials assessing the effectiveness of anti-VEGF agents for the treatment of DME [21,22,23,24]. Nonetheless, there is evidence that high levels of HbA1c at baseline are associated with poor response to intravitreal anti-VEGF therapy [25,26,27]. This is an important point that merits a specific study in which not only baseline HbA1c should be taken into account, but also glycemic variability and the metabolic control after intravitreal anti-VEGF injections. 

Additionally, in the study of Shimura et al. [15] as well as in the present study, mean levels of VEGF in AH were under 200 pg/mL, whereas in the study of Hillier et al. [16] mean VEGF levels were higher than 750 pg/mL. This difference could be attributed to the inclusion of patients with retinal ischemia, a well-recognized factor that upregulates VEGF production, thus limiting the effectiveness of anti-VEGF treatment. The methodological differences could also contribute to the discrepancy of the results. In this regard, it is worth mentioning that we found a strong correlation between the concentration of VEGF in vitreous and aqueous samples obtained simultaneously, thus demonstrating that our samples of AH indeed reflected the events that occurred at retinal level. 

The current body of evidence shows that in patients with type two diabetes, there is a low grade of systemic inflammation, and both adipose tissue and macrophages are thought to be the main sources of systemic proinflammatory cytokine production [28]. Plasma diffusion favored by the breakdown of the BRB in patients with DME could increase the levels of these cytokines in ocular fluids. However, local synthesis is the most important source of proinflammatory cytokines/chemokines involved in DME [11]. Indeed, we have found that levels of VEGF were significantly higher in vitreous fluid than in serum of patients with PDR but not in non-diabetic subjects [29]. We also found that other cytokines such as IL-8 and MCP-1 were strikingly higher in vitreous fluid than in serum of patients with PDR [30]. Notably, the vitreous levels of both IL-8 and MCP-1 were similar to those detected in pleural effusions of patients with pneumonia or tuberculosis. Other authors have reported a significant alteration of inflammatory cytokines in AH and their relationship with the disease severity not only in DME [31] but also in DR [32].

Although AH does not reflect so closely the events that are taking place in the retina as vitreous fluid, it is more easily obtained and therefore “liquid biopsy” of AH could be incorporated into current clinical practice. In the present study we have found that slow responders to anti-VEGF exhibited higher levels of inflammatory biomarkers (IL-6, IL-8, TNFR1, TNFR2 and MMP-9) than rapid responders, thus suggesting that inflammation is not adequately controlled by anti-VEGF agents. In this regard, it has been reported that IL-8 concentration in AH was associated with lower responsiveness to intravitreal bevacizumab [33]. On the other hand, a post-hoc analysis of the READ3 study, in which DME patients were randomized to receive 0.5 or 2.0 mg ranibizumab and AH samples were collected in a prospective manner, has revealed that those patients who presented elevated AH levels of IL-6 at baseline ended up with worse visual outcomes when compared to those with initial low AH IL-6 levels. These data suggest that aqueous IL-6 concentration may have prognostic therapeutic implications, potentially representing a candidate biomarker for a less favorable response to anti-VEGF therapy [34,35,36].

## 5. Conclusions

In conclusion, the use of AH obtained during the first intravitreal injection (“liquid biopsy”) in treatment-naïve DME patients could be useful for determining whether the predominant pathogenic event is the increase of VEGF or other factors. This approach would allow to implement a personalized medicine strategy in terms of selecting a more rational and probably a more cost-effective treatment. Further studies to validate these preliminary results are warranted.

## Figures and Tables

**Table 1:** Coefficient of intra-assay variation and lower detection limits.

	CV Intra-Assay	Low Limit of Detection (pg/mL)
VEGF	0.57%	2.76
PlGF	0.19%	0.05
HGF	0.76%	1.50
EGF	13.85%	2.49
Angiopoietin 2	0.56%	2.41
PDGF-AA	0.65%	0.88
PDGF-AB/BB	0.94%	1.30
MMP-9	0.17%	1.10
IL-1β	1.16%	0.24
IL-6	1.15%	0.78
IL-8	0.51%	1.20
IL-10	0.71%	1.75
IL-12p70	0.59%	1.69
TNF-α	1.02%	1.41
TNFR1	0.36%	4.59
TNFR2	0.55%	9.00

CV: Coefficient of variation; VEGF: Vascular endothelial growth factor; PlGF: placental growth factor; HGF: hepatocyte growth factor; EGF: epidermal growth factor; PDGF: platelet derived growth factor; MMP: matrix metalloproteinase; IL: interleukin; TNF: tumor necrosis factor; TNFR: tumor necrosis factor receptor.

**Table 2:** Characteristics of diabetic patients treated with anti-VEGF agents.

	Rapid-Response to anti-VEGF (*n* = 11)	Slow-Response to anti-VEGF (*n* = 11)	Non-Response to anti-VEGF (*n* = 9)	*p*-Value
Gender (M/F)	6/5	6/5	7/2	n.s.
Age (years)	67.5 ± 9.7	71 ± 6.9	68.1 ± 11.6	n.s.
Diabetes duration (years)	12.7 ± 2.6	12.9 ± 2.6	11.6 ± 2.9	n.s.
HbA1c (%)	7.75 ± 1.34	7.54 ± 1.11	7.84 ± 1.3	n.s.
Insulin treatment (%)	70	81.8	33.3	n.s.
Phakic status (%)				n.s.
No cataract	4	2	5	
Cataract	0	1	0	
Previous cataract surgery	6	8	4	
Glaucoma (%)	10	27.3	33.3	n.s.
NPDR (%) / PDR (%)	91 / 9	82/18	89/11	n.s.
Central Retinal Thickness (µm)	443 ± 91	542 ± 179	449 ± 67	n.s.
Macular volume (mm^3^)	9.67 ± 1.83	11.55 ± 4.16	9.62 ± 1.37	n.s.
Mean BCVA (ETDRS letters)	65 (20/50)	67.2 (20/50)	62.5 (20/63)	n.s.

VEGF: Vascular Endothelial Growth Factor; M/F: Male, female; NPDR: non-proliferative diabetic retinopathy; PDR: proliferative diabetic retinopathy. BCVA: best corrected visual acuity. ETDRS: Early Treatment Diabetic Retinopathy Study.

**Table 3:** Concentration of growth factors and cytokines in aqueous humor (AH) according to anti-VEGF treatment response.

	Rapid-Response to anti-VEGF (*n* = 11)	Delayed-Response to anti-VEGF (*n* = 11)	Non-Response to anti-VEGF (*n* = 9)	*p*-Value
Group	1	2	3	
VEGF (pg/mL)	62.2 (10–160)	10.1 (2.7–26.8)	2.7 (2.7–55)	0.08 *
				0.03 **
PIGF (pg/mL)	266 (3.8–266)	266 (8.9–266)	266 (2.6–266)	0.84 *
				0.99 **
HGF (pg/mL)	343 (35.19–433)	482 (198–2036)	258 (177–759)	0.15 *
				0.94 **
Angiopoietin-2 (pg/mL)	5.6 (4.5–20.7)	15.5 (5.6–30.62)	6.8 (2–21.9)	0.13 *
				0.88 **
PDGF-AA (pg/mL)	18.9 (6.8–18.9)	27.2 (22–27.3)	13 (11.2–17.5)	0.74 *
				0.37 **
MMP-9 (pg/mL)	74.3 (28.4–174)	237 (93.4–282)	73 (2–182)	0.07 *
				0.41 **
IL-6 (pg/mL)	4 (0.7–17)	6.7 (3–13.6)	1.52 (0.7–7.7)	0.51 *
				0.71 **
IL-8 (pg/mL)	3.2 (1.6–10.6)	11.5 (4.1–25.8)	4.9 (2.3–12.4)	0.07*
				0.60 **
TNFR1 (pg/mL)	122 (32.5–168)	171 (91.4–307)	114 (46.5–219)	0.10 *
				0.90 **
TNFR2 (pg/mL)	215 (85.3–372)	340 (210–755)	234 (124–352)	0.07 *
				0.76 **

Data are median (CI 25%–75%). *: *p*-value between group 1 and group 2, **: *p*-value between group 1 and group 3. VEGF: Vascular endothelial growth factor; PlGF: placental growth factor; HGF: hepatocyte growth factor; PDGF: platelet derived growth factor; MMP: matrix metalloproteinase; IL: interleukin; TNF: tumor necrosis factor; TNFR: tumor necrosis factor receptor.

## References

[1] Yau J.W., Rogers S.L., Kawasaki R., Lamoureux E.L., Kowalski J.W., Bek T., Chen S.J., Dekker J.M., Fletcher A., Grauslund J. (2012). Global prevalence and major risk factors of diabetic retinopathy. Diabetes Care.

[2] Lightman S., Towler H.M. (2003). Diabetic retinopathy. Clin. Cornerstone.

[3] Tong L., Vernon S.A., Kiel W., Sung V., Orr G.M. (2001). Association of macular involvement with proliferative retinopathy in type 2 diabetes. Diabet. Med..

[4] Simó R., Hernández C. (2015). Novel approaches for treating diabetic retinopathy based on recent pathogenic evidence. Prog. Retin. Eye Res..

[5] Tan G.S., Cheung N., Simó R., Cheung G.C., Wong T.Y. (2017). Diabetic macular oedema. Lancet Diabetes Endocrinol..

[6] Cheung N., Wong I.Y., Wong T.Y. (2014). Ocular anti-VEGF therapy for diabetic retinopathy: Overview of clinical efficacy and evolving applications. Diabetes Care.

[7] Simó R., Sundstrom J.M., Antonetti D.A. (2014). Ocular Anti-VEGF therapy for diabetic retinopathy: The role of VEGF in the pathogenesis of diabetic retinopathy. Diabetes Care.

[8] Virgili G., Parravano M., Menchini F., Evans J.R. (2014). Anti-vascular endothelial growth factor for diabetic macular oedema. Cochrane Database Syst. Rev..

[9] Gross J.G., Glassman A.R., Jampol L.M., Inusah S., Aiello L.P., Antoszyk A.N., Baker C.W., Berger B.B., Bressler N.M., Browning D. (2015). Panretinal Photocoagulation vs Intravitreous Ranibizumab for Proliferative Diabetic Retinopathy: A Randomized Clinical Trial. JAMA.

[10] Aiello L.P., Avery R.L., Arrigg P.G., Keyt B.A., Jampel H.D., Shah S.T., Pasquale L.R., Thieme H., Iwamoto M.A., Park J.E. (1994). Vascular endothelial growth factor in ocular fluid of patients with diabetic retinopathy and other retinal disorders. N. Engl. J. Med..

[11] Simó-Servat O., Hernández C., Simó R. (2012). Usefulness of the vitreous fluid analysis in the translational research of diabetic retinopathy. Mediat. Inflamm..

[12] Funatsu H., Yamashita H., Noma H., Mimura T., Nakamura S., Sakata K., Hori S. (2005). Aqueous humor levels of cytokines are related to vitreous levels and progression of diabetic retinopathy in diabetic patients. Graefe’s Arch. Clin. Exp. Opthalmol..

[13] Noma H., Funatsu H., Yamasaki M., Tsukamoto H., Mimura T., Sone T., Hirayama T., Tamura H., Yamashita H., Minamoto A. (2008). Aqueous humour levels of cytokines are correlated to vitreous levels and severity of macular oedema in branch retinal vein occlusion. Eye.

[14] Vujosevic S., Simó R. (2017). Local and Systemic Inflammatory Biomarkers of Diabetic Retinopathy: An Integrative Approach. Investig. Ophthalmol. Vis. Sci..

[15] Shimura M., Yasuda K., Motohashi R., Kotake O., Noma H. (2017). Aqueous cytokine and growth factor levels indicate response to ranibizumab for DME. Br. J. Ophthalmol..

[16] Hillier R.J., Ojaimi E., Wong D.T., Mak M.Y.K., Berger A.R., Kohly R.P., Kertes P.J., Forooghian F., Boyd S.R., Eng K. (2018). Aqueous Humor Cytokine Levels and Anatomic Response to Intravitreal Ranibizumab in Diabetic Macular Edema. JAMA Ophthalmol..

[17] Nisic F., Jovanovic N., Mavija M., Alimanovic-Halilovic E., Nisic A., Lepara O., Cemerlic A. (2019). Vitreous concentrations of vascular endothelial growth factor as a potential biomarker for postoperative complications following pars plana vitrectomy. Arch. Med. Sci..

[18] Boyer D.S., Yoon Y.H., Belfort R., Bandello F., Maturi R.K., Augustin A.J., Li X.Y., Cui H., Hashad Y., Whitcup S.M. (2014). Three-year, randomized, sham-controlled trial of dexamethasone intravitreal implant in patients with diabetic macular edema. Ophthalmology.

[19] Ankor R., Yonekawa Y., Todorich B., Van Laere L., Hussain R., Woodward M.A., Abbey A.M., Wolfe J.D. (2017). Prediction of Anti-VEGF Response in Diabetic Macular Edema After 1 Injection. J. Vitreoretin. Dis..

[20] Gonzalez V.H., Campbell J., Holekamp N.M., Kiss S., Loewenstein A., Augustin A.J., Ma J., Ho A.C., Patel V., Whitcup S.M. (2016). Early and Long-Term Responses to Anti-Vascular Endothelial Growth Factor Therapy in Diabetic Macular Edema: Analysis of Protocol I Data. Am. J. Ophthalmol..

[21] Thomas B.J., Shienbaum G., Boyer D.S., Flynn H.W. (2013). Evolving strategies in the management of diabetic macular edema: Clinical trials and current management. Can. J. Ophthalmol..

[22] Brown D.M., Nguyen Q.D., Marcus D.M., Boyer D.S., Patel S., Feiner L., Schlottmann P.G., Rundle A.C., Zhang J., Rubio R.G. (2013). Long-term outcomes of ranibizumab therapy for diabetic macular edema: The 36-month results from two phase III trials: RISE and RIDE. Ophthalmology.

[23] Schmidt-Erfurth U., Lang G.E., Holz F.G., Schlingemann R.O., Lanzetta P., Massin P., Gerstner O., Bouazza A.S., Shen H., Osborne A. (2014). Three-year outcomes of individualized ranibizumab treatment in patients with diabetic macular edema: The RESTORE extension study. Ophthalmology.

[24] Bressler N.M., Beaulieu W.T., Maguire M.G., Glassman A.R., Blinder K.J., Bressler S.B., Gonzalez V.H., Jampol L.M., Melia M., Sun J.K. (2018). Diabetic Retinopathy Clinical Research Network. Early Response to Anti-Vascular Endothelial Growth Factor and Two-Year Outcomes among Eyes with Diabetic Macular Edema in Protocol T. Am. J. Ophthalmol..

[25] Macky T.A., Mahgoub M.M. (2012). The effect of glycemic control on visual and anatomic outcomes in response to therapy for diabetic macular edema. Eur. J. Ophthalmol..

[26] Matsuda S., Tam T., Singh R.P., Kaiser P.K., Petkovsek D., Carneiro G., Zanella M.T., Ehlers J.P. (2014). The impact of metabolic parameters on clinical response to VEGF inhibitors for diabetic macular edema. J. Diabetes Complicat..

[27] Kim T.K., Shin H.Y., Kim S.Y., Lee Y.C., Lee M.Y. (2017). Factors Influencing Intravitreal Bevacizumab and Triamcinolone Treatment in Patients with Diabetic Macular Edema. Eur. J. Ophthalmol..

[28] Donath M.Y., Shoelson S.E. (2011). Type 2 diabetes as an inflammatory disease. Nat. Rev. Immunol..

[29] Simó R., Vidal M.T., García-Arumí J., Carrasco E., García-Ramírez M., Segura R.M., Hernández C. (2006). Intravitreous hepatocyte growth factor in patients with proliferative diabetic retinopathy: A case-control study. Diabetes Res. Clin. Pract..

[30] Hernández C., Segura R.M., Fonollosa A., Carrasco E., Francisco G., Simó R. (2005). Interleukin-8, monocyte chemoattractant protein-1 and IL-10 in the vitreous fluid of patients with proliferative diabetic retinopathy. Diabet. Med..

[31] Hillier R.J., Ojaimi E., Wong D.T., Mak M.Y., Berger A.R., Kohly R.P., Kertes P.J., Forooghian F., Boyd S.R., Eng K. (2017). Aqueous humor cytokine levels as biomarkers of disease severity in diabetic macular edema. Retina.

[32] Chen H., Zhang X., Liao N., Wen F. (2017). Assessment of biomarkers using multiplex assays in aqueous humor of patients with diabetic retinopathy. BMC Ophthalmol..

[33] Kwon J.W., Jee D. (2018). Aqueous humor cytokine levels in patients with diabetic macular edema refractory to anti-VEGF treatment. PLoS ONE.

[34] Sepah Y.J., Nguyen Q.D., Do D.V., Day B., Wakshull E., Stoilov I. Trends for poorer vision outcomes in nAMD and DME patients with higher aqueous humor levels of IL-6. Proceedings of the Association for Research in Vision and Ophthalmology’s (ARVO) Annual Meeting.

[35] Affridi R., Halim M.S., Sadiq M.A., Hassan M., Agarwal A., Do D.V., Nguyen Q.D., Sepah J. Can the levels of inflammatory cytokines in the anterior chamber of eyes with diabetic macular edema predict response to therapy?. Proceedings of the Association for Research in Vision and Ophthalmology’s (ARVO) Annual Meeting.

[36] Chalam K.V., Grover S., Sambhav K., Balaiya S., Murthy R.K. (2014). Aqueous interleukin-6 levels are superior to vascular endothelial growth factor in predicting therapeutic response to bevacizumab in age-related macular degeneration. J. Ophthalmol..

